# Amantadine-induced reorganization of model SARS-CoV-2 lipid envelopes

**DOI:** 10.1039/d6ra02249a

**Published:** 2026-07-21

**Authors:** Marta Mierzejewska, Izabella Leszczyńska, Gabriela Węsierska, Piotr Batys, Dorota Matyszewska

**Affiliations:** a University of Warsaw, Faculty of Chemistry, Biological and Chemical Research Centre Żwirki i Wigury 101 02089 Warsaw Poland dorota.matyszewska@chem.uw.edu.pl; b Jerzy Haber Institute of Catalysis and Surface Chemistry, Polish Academy of Sciences Niezapominajek 8 30239 Krakow Poland

## Abstract

Amantadine attracted renewed interest during the COVID-19 pandemic because of its known antiviral activity against influenza A. In this work, we investigated how amantadine affects a simplified model of the SARS-CoV-2 lipid envelope composed of DOPC : DMPS : PI (50 : 35 : 15), as well as monolayers of the individual lipids: DOPC, DMPS and PI. Langmuir experiments showed that the effect of the drug depends on the lipid type. In DOPC and PI monolayers, amantadine increased the area per molecule, suggesting its incorporation into more fluid layers. In contrast, in more compact DMPS monolayers, stronger electrostatic interactions led to different behaviour and promoted tighter packing at higher surface pressures. For the ternary model, excess area and compression–expansion hysteresis analyses pointed to drug-induced domain formation and changes in monolayer organization, which were confirmed by Brewster angle microscopy. To extend these results to 3D systems, liposomes and giant unilamellar vesicles were used as bilayer models. Dynamic light scattering revealed changes in hydrodynamic diameter and zeta potential, while fluorescence microscopy confirmed amantadine incorporation together with bilayer reorganization. Molecular dynamics simulations supported the experimental observations and showed that amantadine preferentially locates at the interface and partially inserts into the lipid layer. Overall, the results show that amantadine interactions with simplified SARS-CoV-2 lipid envelopes depend on both the net charge of the lipid headgroup and the membrane organization, which is influenced by the presence or absence of unsaturated alkyl chains in the hydrophobic region.

## Abstract 2

Amantadine, an antiviral agent against influenza A, may affect physicochemical properties of 2D and 3D models of viral lipid envelope of SARS-CoV-2 virus.

## Graphical abstract

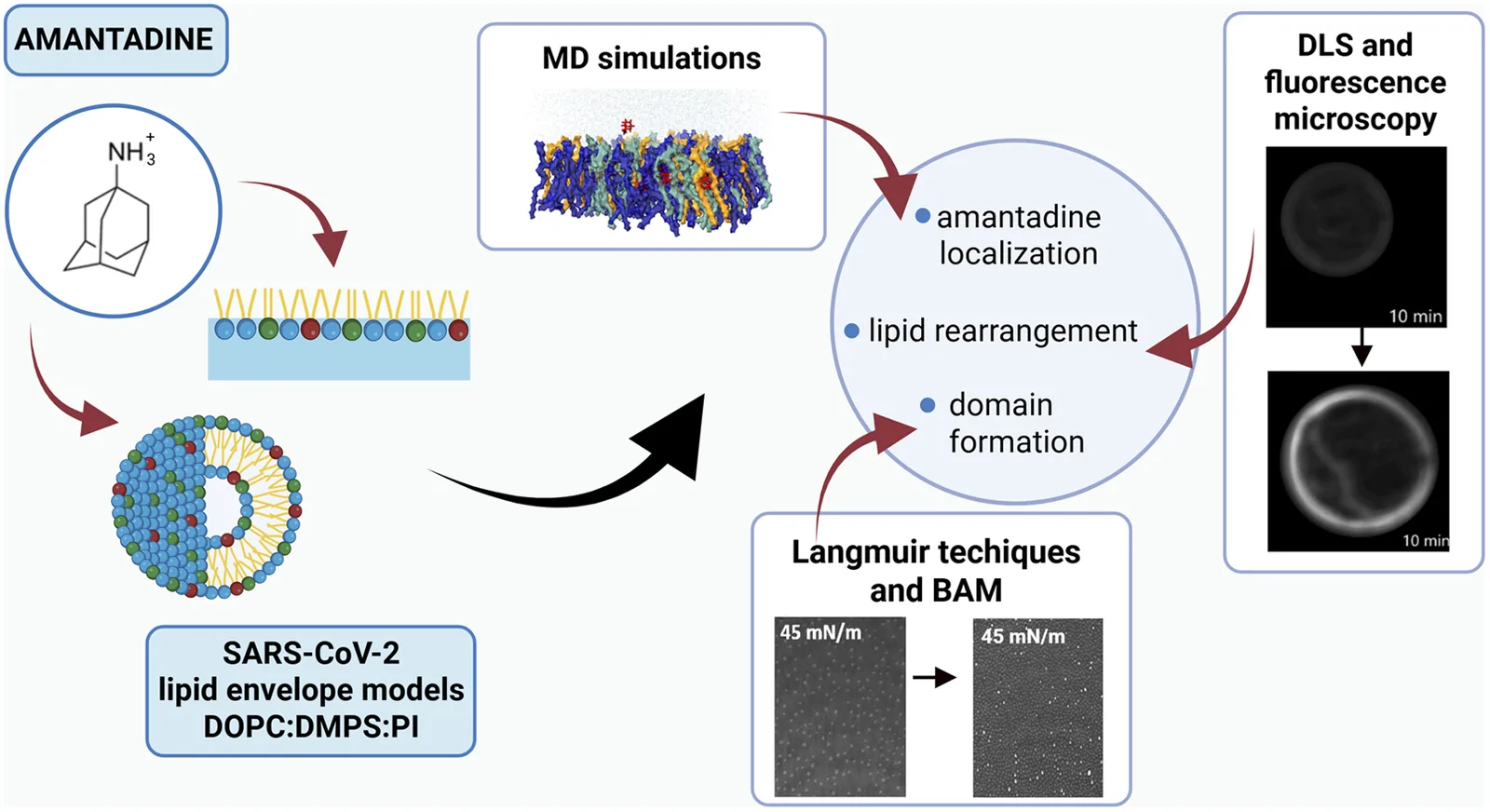

## 1. Introduction

SARS-CoV-2 (Severe Acute Respiratory Syndrome Coronavirus 2) is a novel β-coronavirus that was first identified in 2019, and caused a worldwide pandemic, declared by the WHO in 2020.^1–3^ Due to its ability to spread easily through droplets and contaminated surfaces, the virus caused millions of deaths worldwide.^3^ As an enveloped virus, it consists of a lipid coat surrounding a protein capsid that encloses viral genetic material and ensures the virus' stability and protection from external factors. During the pandemic, scientists investigated existing antiviral drugs to combat COVID-19.^4–6^ One potentially promising candidate was amantadine – a drug conventionally used in the treatment of Parkinson's disease and influenza A virus infections.^7^

**Fig. 1:**
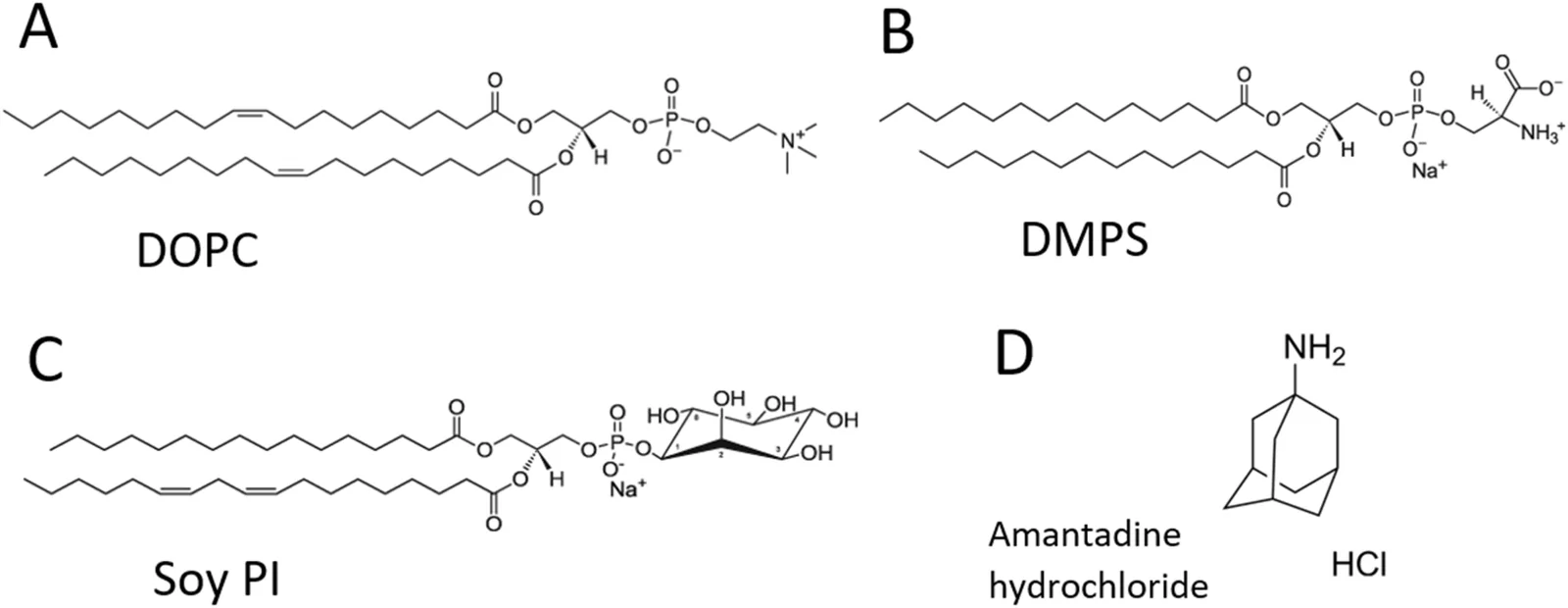
Structures of: (A) DOPC; (B) DMPS; (C) PI; (D) amantadine hydrochloride.

Amantadine is a small amphiphilic compound, composed of adamantane backbone and amino group (Fig. 1). As an antiviral agent used against influenza infection, it inhibits the M2 protein (ion channel) by blocking H^+^ ion transport and inducing its structural changes,^7^ which prevents the release of viral genetic material required for replication.^8,9^ Furthermore, amantadine has been found to inhibit lipid membrane fusion;^10^ when the M2 channel is saturated with the drug, excess molecules can interact directly with the surrounding membrane lipids.^11^ By 2009, amantadine was no longer recommended as an antiviral agent due to rising resistance in new influenza strains, but the COVID-19 pandemic sparked a renewed interest in its mechanisms. Recent studies suggest that amantadine affects the function of the SARS-CoV-2 E protein, complicating the release of viral genetic material.^12,13^ This hypothesis is supported by research demonstrating its ability to inhibit viral replication in Vero E6 cells.^14^ Although some papers report no effect of amantadine on COVID-19 treatment,^15,16^ others indicate beneficial outcomes, particularly when administered early in the disease course.^17–19^ This inconsistency highlights the need for further studies on amantadine's mechanism of action in the context of COVID-19.

Numerous studies have investigated interactions of amantadine with lipid membranes. Already in 1984 Tverdislov *et al.* demonstrated these interactions by tracking changes in the elasticity and conductance of azolectin membranes exposed to amantadine.^20^ Subsequent neutron and X-ray diffraction studies confirmed that amantadine alters the arrangement and translational diffusion of DOPC molecules, with its hydrocarbon core positioned near the phosphate and ester groups, and its amino group oriented toward the aqueous phase.^21^ In another study, researchers demonstrated that protonated amantadine exhibits a greater potential for membrane incorporation than its deprotonated form. Saturation Transfer Difference (STD) NMR results identified the glycerol CH_2_ groups located near the negatively charged phosphate groups in PC lipids as the primary regions where amantadine localizes. It was also noted that amantadine is not permanently bound to the lipids and retains mobility within the bilayer.^10^

In POPC membranes, the amantadine backbone embeds in the alkyl chain region, while the amino group interacts with the oxygen atoms of the choline and glycerol moieties.^22^ Moreover, solid-state NMR and molecular dynamics (MD) simulations revealed that amantadine adopts a preferred equilibrium orientation in the lipid layer. When localized within DMPC layers, it becomes entangled with the lipids, leading to a reoriented packing that distorts the bilayer.^23^ An interesting study by Wu *et al.* highlighted the differences in amantadine's interactions with PC and PG lipids, driven by their differing polar headgroup charges. At low concentrations (up to 5 mM), the drug does not disrupt DPPC layer integrity, but it significantly affects DPPG through attractive electrostatic interactions. However, at concentrations up to 10 mM, amantadine aggregates in both layers, leading to bilayer disruption.^7^ Additionally, amantadine penetrates highly hydrated DMPC layers more easily than DMPE layers, which are tightly packed due to their smaller headgroups. In DMPC layers, changes in integrity occur in both the hydrocarbon and headgroup regions, indicating amantadine's ability to disrupt electrostatic and hydrogen bonds within the lipid matrix.^24^

While these studies provide valuable insights into amantadine-membrane interactions, the existing literature predominantly focuses on very simplified, single-component lipid models comprising mostly zwitterionic lipids. To date, the biophysical impact of the drug on the SARS-CoV-2 virus envelope of the specific lipid composition has not yet been thoroughly investigated. To address this, our study investigates the physicochemical interactions between amantadine and a more precisely adjusted model of the SARS-CoV-2 lipid envelope. We utilized a DOPC : DMPS : PI (50 : 35 : 15 molar ratio) composition (Fig. 1) to reflect the primary components of the coronavirus lipid envelope,^25,26^ allowing us to evaluate the electrostatic interactions between the drug and the membrane. To obtain a comprehensive understanding of these interactions, we combined macroscopic and microscopic experimental techniques with molecular modelling. We analysed 2D planar models (Langmuir monolayers) using Langmuir technique combined with Brewster angle microscopy (BAM), and 3D spherical models (liposomes) *via* dynamic light scattering (DLS) and fluorescence microscopy. These experimental results were complemented by MD simulations to predict amantadine localization and behaviour at the molecular level. By systematically comparing this ternary system against single-component monolayers, our research aims to clarify the biophysical mechanisms underlying amantadine-induced disruption of viral lipid envelopes with a composition specifically adjusted to mimic SARS-CoV-2 virus envelope. Knowledge of the preferential interactions with specific phospholipids of the viral envelope may be useful for further investigation on processes that are crucial for infectivity of the virus, such as membrane fusion or protein embedding within the lipid membrane. Additionally, such an approach of targeting viral lipid envelopes instead of proteins relies on the interactions with host-derived phospholipids, which are independent of viral genetic variability and therefore allows for the prevention of resistance to amantadine, which was observed previously in the case of influenza treatment.

## 2. Experimental

### 2.1. Materials

DOPC (1,2-dioleoyl-*sn*-glycero-3-phosphocholine), DMPS (1,2-dimyristoyl-*sn*-glycero-3-phospho-l-serine (sodium salt)) and PI (l-α-phosphatidylinositol (sodium salt) (Soy extract)) as components of SARS-CoV-2 viral lipid envelope model were purchased from Avanti Polar Lipids (USA) and used without further purification. Amantadine hydrochloride was bought from Sigma-Aldrich (Darmstadt, Germany). The fluorescent probe used in GUVs visualization, NBD-PC (phosphatidylcholine probe), was bought from Avanti Polar Lipids (USA). During all experiments, ultrapure Milli-Q water with a resistivity of 18.2 MΩ was used. All lipid solutions used in Langmuir technique were prepared in a mixture of organic solvents chloroform : methanol 4 : 1 (v/v). Single-component lipid solutions were prepared at a concentration of 1 mg mL^−1^, and the ternary DOPC : DMPS : PI solution at a molar ratio of 50 : 35 : 15 was prepared at a concentration of 1 µmol mL^−1^.

### 2.2. Methods

#### 2.2.1. Langmuir technique

Changes in the physicochemical properties of Langmuir lipid monolayers were monitored as changes in surface pressure at the air–water interface using a KSV Nima Langmuir trough with dimensions 78.4 × 74 cm (580 cm^2^), two hydrophilic barriers moving at the surface of the subphase, and a Wilhelmy microbalance equipped with filter paper of specified dimensions. The trough was placed inside a closed cabinet to prevent contamination of the measurement system and on an anti-vibration table to minimize movements that could influence the surface pressure measurements. At the beginning of each measurement, the hydrophobic trough surface was cleaned with chloroform, methanol and MiliQ water, while the hydrophilic barriers were cleaned with methanol and MiliQ water. Lipid solutions were spread onto the previously cleaned water subphase using a Hamilton microsyringe (with an accuracy of 0.5 µl). Compression of the monolayer began 10 minutes after spreading, allowing sufficient time for evaporation of the organic solvents and adsorption of lipids. During the measurements, the barriers moved at a speed of 10 mm min^−1^, and the surface pressure was recorded (with an accuracy of 0.1 mN m^−1^), and plotted as the surface pressure–area per molecule (π–*A*) isotherms. All experiments were conducted at 21 °C.

The elastic properties of the formed lipid layers can be described based on the compression modulus (*C*_s_^−1^), which is determined according to the following equation:^27,28^1
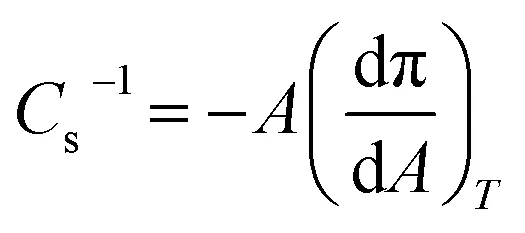
where *C*_s_^−1^ is the compression modulus, *A* represents the area per molecule, and π is the surface pressure. The maximum values of the compression modulus provide insight into the phase state of the monolayer. Depending on the *C*_s max_^−1^ value, the monolayer can exist in the gaseous phase (G, 0–12.5 mN m^−1^), the liquid-expanded phase (LE, 12.5–50 mN m^−1^), the liquid-condensed phase (LC, 100–250 mN m^−1^), or the solid phase (S, above 250 mN m^−1^).^29^ The presence of minima in the compression modulus *versus* surface pressure (*C*_s_^−1^–π) plot indicates a phase transition or partial collapse of the monolayer.

A cyclic compression–decompression of a ternary monolayer was carried out within the surface pressure range of 0–30 mN m^−1^. Obtained isotherms allow to observe hysteresis in the physicochemical properties of the lipid layer. Based on this cyclic process, thermodynamic parameters, such as the free energy of compression/expansion (Δ*G*_comp/exp_), the free energy of hysteresis (Δ*G*^hys^), the configurational entropy of hysteresis (Δ*S*^hys^) and the enthalpy of hysteresis (Δ*H*^hys^) were calculated using the following equations:^30–33^2
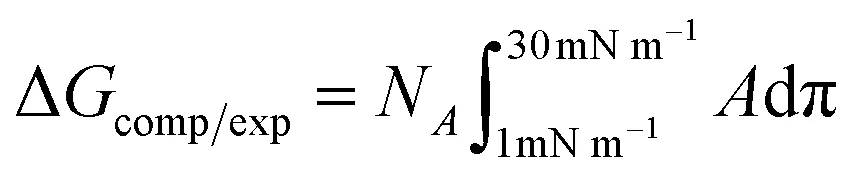

3 Δ*G*^hys^ = Δ*G*_exp_ − Δ*G*_comp_

4
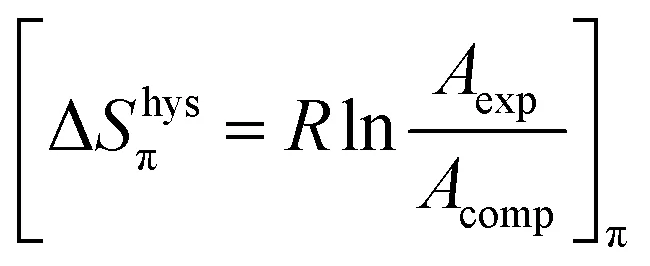
5
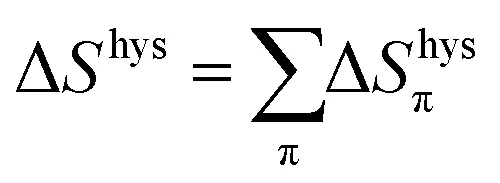

6 Δ*H*^hys^ = Δ*G*^hys^ + *T*Δ*S*^hys^

where *A*_comp_ and Δ*G*_comp_ is the area and energy of the compression, *A*_exp_ and Δ*G*_exp_ is the area and energy of the decompression, *N*_A_ is the Avogadro number, *R* is the gas constant and *T* is temperature.

The other type of Langmuir experiment involved compressing the monolayers to a target surface pressure of 30 mN m^−1^, at which the monolayer properties resemble the properties of bilayers^34,35^ using a barrier speed of 10 mm min^−1^ (7.5 cm^2^ min^−1^). While maintaining this constant pressure, the variation in monolayer area caused by amantadine interaction was recorded. The data was analysed as the relative area change (*A/A*_0_) over time, where *A*_0_ represents the initial area recorded immediately upon reaching the target pressure.

#### 2.2.2. Brewster angle microscopy (BAM)

To visualize changes of lipid monolayers morphology during the compression, the images of its surface were captured using Brewster Angle Microscopy (BAM). In this case Nanofilm Ep3 setup and UltraBAM objective (Accurion, Germany) were used to get images with 800 µm × 430 µm field of view and with lateral resolution of 2 µm. High-quality images showing differences in lipid organization and its consequences at the air–water interface were obtained.

#### 2.2.3. Liposomes preparation

Liposomes as three-dimensional bilayer models were prepared using the Bangham method (hydration of thin lipid film). Lipids were dissolved in a chloroform : methanol 4 : 1 (v/v) mixture to obtain single-component solutions with a final concentration of 5 mg mL^−1^. Then the appropriate volumes were mixed to prepare a ternary mixture of DOPC : DMPS : PI at a 50 : 35 : 15 molar ratio. Equal volumes of the solution were transferred into Eppendorf tubes and the solvent was evaporated under a stream of argon. Each tube was secured with a layer of parafilm to protect the unsaturated alkyl chains from oxidation upon exposure to air.^36,37^ The lipid films were placed in a vacuum desiccator for 2 h to remove residual organic solvents, then hydrated with 0.5 mL of PBS buffer at pH 7.4 and sonicated at 37 °C for 1.5 h for liposome formation.^38^ Before measurements, the hydrated liposomes were stored for 24 h in a refrigerator at 3 °C. The remaining Eppendorf tubes containing dry lipid films were stored in a freezer at −4 °C prior to the hydration. Immediately before measurement, the liposomes were extruded fifteen times through 100 nm polycarbonate membranes to achieve homogeneous liposome size.

#### 2.2.4. Hydrodynamic diameter and zeta potential measurements of liposomes

The hydrodynamic diameter and zeta potential of liposomes in PBS buffer (pH 7.4) were measured using a Zetasizer Nano ZSP (Malvern Panalytical, Malvern, UK). Measurements were performed at a fixed scattering angle of 173°, using Malvern software. All calculations were based on the Helmholtz–Smoluchowski equation.^39^ For hydrodynamic diameter and zeta potential measurements, liposome suspensions in PBS (pH 7.4) were prepared at concentrations of 0.05 mg mL^−1^ and 0.0025 mg mL^−1^, respectively. Prior to insertion into the instrument, the samples were mixed by hand for one minute to assure homogeneity of the sample. For hydrodynamic diameter measurements, quartz cuvettes were used and dedicated zeta potential cuvettes equipped with two electrodes (Malvern Panalytical, Malvern, UK) were employed for zeta potential analysis. Before each measurement, the solution placed in the cuvette was equilibrated at 25 °C for 2 minutes. Measurement proceeded for 120 minutes, with data collected at intervals optimally determined by the software dependent on the sample.

#### 2.2.5. Giant unilamellar vesicles (GUV)s preparation and visualizatiom

GUVs were formed using the electroformation method.^40^ First, single-component lipid solutions were prepared in a chloroform : methanol 4 : 1 (v/v) mixture and then combined in specific volumes to obtain a ternary lipid solution of DOPC : DMPS : PI (50 : 35 : 15 molar ratio), at a concentration of 10 mg mL^−1^ with addition of 1% fluorescent probe NBD-PC. The solution was transferred into an electroformation chamber equipped with two platinum electrodes^41^ and diluted with chloroform so that the solution covered the entire electrode surface. The top of the chamber was secured with parafilm to prevent oxidation of the unsaturated alkyl chains upon air exposure, then organic solvent was evaporated under a stream of argon. The deposited lipid film was dried in a vacuum desiccator for 2 hours to remove residual organic solvents. After this period, a 200 mmol L^−1^ sucrose solution prepared in PBS buffer (pH 7.4) was added to the chamber, the electrodes were connected to a potentiostat, and an alternating current (AC) electric field (10 Hz, 1.0 V amplitude) was applied for 1.5 hours.^42^ The obtained liposomes were diluted in a 200 mmol L^−1^ glucose solution in PBS (pH 7.4)^43^ to obtain a final lipid concentration of 0.0075 mg mL^−1^ and placed into Millicell EZ SLIDE 8-well glass chambers (Merck KGaA, Darmstadt, Germany). Liposomes were observed under an optical microscope (Nikon Eclipse Ni-U, Tokyo, Japan) for 30 minutes, each system was observed three times. Images of the liposomes were acquired using JENOPTIK GRYPHAX software.

#### 2.2.6. Molecular dynamics simulations

All-atom molecular dynamics simulations were performed to investigate the interaction of amantadine with lipid monolayers. Four distinct lipid composition systems were simulated: a ternary monolayer composed of DOPC, DMPS, and PI at a 50 : 35 : 15 molar ratio, and three single-component monolayers consisting of DMPS, DOPC, or PI. Each system was simulated both in the presence and absence of amantadine, with the drug-free systems serving as references. Lipid layers and initial configurations, as well as amantadine topology and parameters, were generated by CHARMM-GUI.^44–48^ Simulations were carried out with GROMACS (version 2024.2),^49–51^ using CHARMM forcefield^52,53^ and the TIP3P water model.^54^ Counterions, Na^+^ and Cl^−^, were added as required to neutralize the systems. The simulation box dimensions were approximately 8 × 8 × 36 nm. Periodic boundary conditions were applied in all directions.

Simulations were conducted for 400 ns with an integration time step of 2 fs. Trajectory frames used for analysis were taken from the completed production runs (coordinates were written every 10 000 steps). Temperature was maintained at 294.15 K using the V-rescale thermostat with coupling time constant of 1.0 ps.^55^ Pressure had been controlled *via* C-rescale barostat using the surface-tension coupling scheme. A constant surface pressure of 30 mN m^−1^ was applied in all simulations, reflecting experimentally relevant lateral pressure conditions of viral lipid bilayers. The Lennard-Jones potential with a 1 nm cutoff was used to describe van der Waals interactions between atoms. Long-range electrostatics were treated with the particle-mesh Ewald (PME) method.^56,57^ Bonds between hydrogen and heavy atoms were constrained by the LINCS algorithm^58^ and water by the SETTLE algorithm.^59^

Coulomb and Lennard-Jones interaction energies between amantadine and lipids were extracted from the short-range energy terms recorded by GROMACS and were reported as time-averaged values over the analysis window. Time series for energy were post-processed using a Savitzky–Golay filter to aid visual interpretation (number of contacts: window = 471 frames, polynomial order = 3; short-range energy traces: window = 951 frames, polynomial order = 3). These smoothing operations were applied solely for figure presentation and do not affect the conclusions reported. Mass density distributions along the membrane *z*-coordinate and radial distribution functions were computed with GROMACS tools *gmx density* and *gmx rdf*, respectively. The excess area was obtained by averaging the *xy* box area over the last 50 ns from production run and normalizing it by the number of lipids. Visualizations were prepared by UCSF ChimeraX program^60–62^ and VMD software.^63^

## 3. Results

### 3.1. Monolayers at the air–water interface as models of viral lipid envelopes

**Fig. 2:**
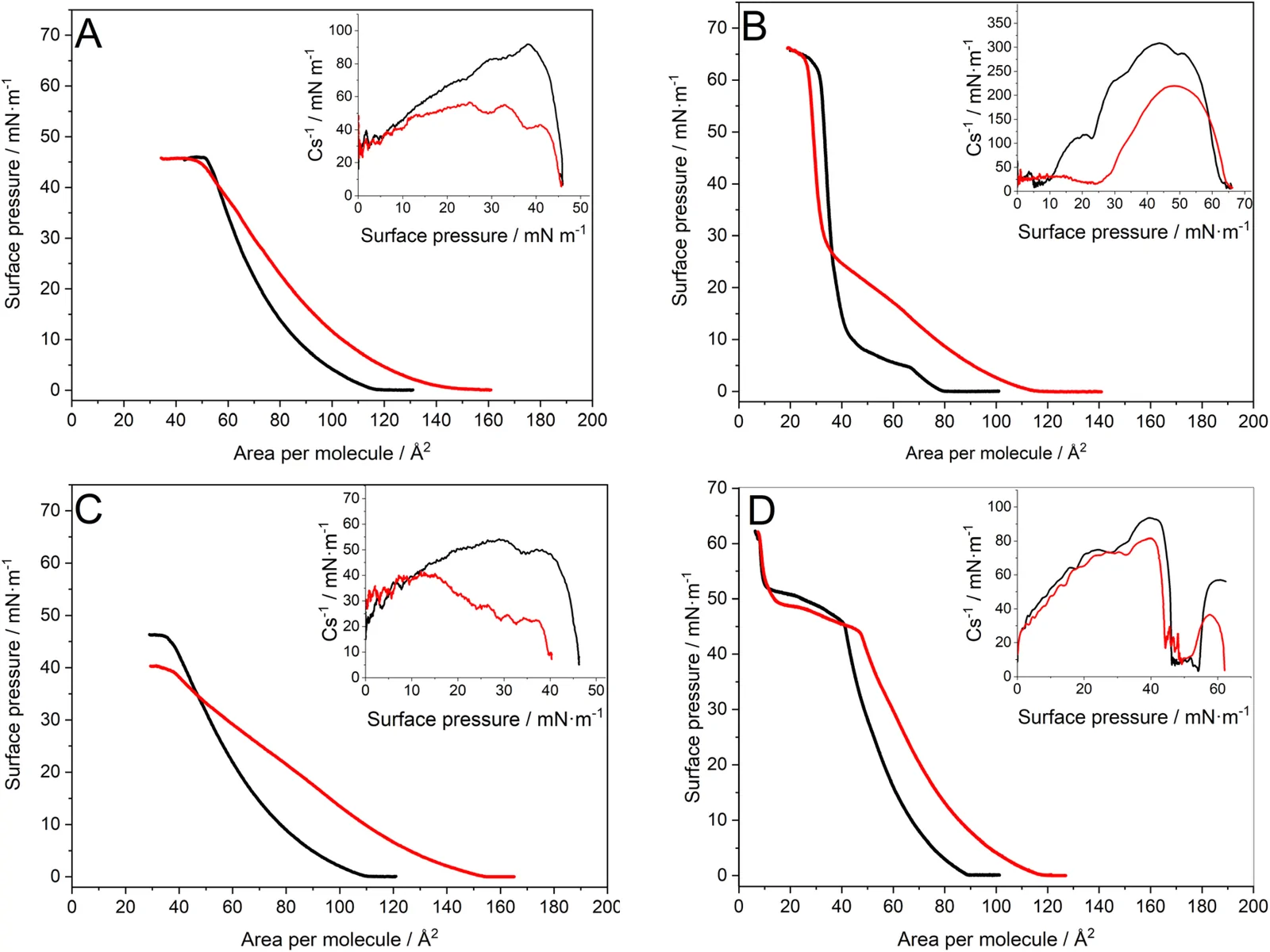
Surface pressure–area per molecule (π–*A*) isotherms of single components: (A) DOPC; (B) DMPS; (C) PI; (D) mixed DOPC : DMPS : PI (50 : 35 : 15) (SARS-CoV-2 model) monolayers on a pure water subphase and subphase containing 10^−5^ mol L^−1^ amantadine. Inset: compression modulus *versus* surface pressure (*C*_s_^−1^–π) plots.

Three single-component lipid monolayers were prepared using the main components of the lipid envelope of the SARS-CoV-2 virus: DOPC, DMPS, and PI, as well as their mixture DOPC : DMPS : PI in a molar ratio of 50 : 35 : 15 chosen on the basis of the envelope composition and previous studies.^25,26^ Each monolayer was compressed on a pure water subphase and then on a water subphase with amantadine at a concentration of 10^−5^ mol L^−1^. On the pure water subphase DOPC and PI monolayers in the maximum compression form stable films in the liquid-expanded (LE) phase, reaching maximum compressibility modulus values (*C*_s max_^−1^) of 88 mN m^−1^ and 54 mN m^−1^, respectively (Fig. 2 and Table 1).^64^ DMPS monolayer forms a solid phase (S) with a *C*_s max_^−1^ value of 293 mN m^−1^ and the presence of a typical phase transition for the DMPS monolayer at a surface pressure of approximately 5 mN m^−1^ is also observed. In the case of the ternary monolayer, it is at the border between liquid-condensed (LC) and liquid phase as reflected by a *C*_s max_^−1^ value of 105 mN m^−1^ (Table 1). Additionally, a partial collapse seen as a plateau on the isotherm and confirmed by the *C*_s_^−1^ = 0 at a surface pressure of approximately 55 mN m^−1^ can also be observed.^65^

**Table 1:** Characteristic parameters of Langmuir monolayers of single phospholipid components and ternary mixed DOPC : DMPS : PI (50 : 35 : 15) monolayers used as model SARS-CoV-2 lipid envelope formed on pure water subphase and subphase with amantadine

Subphase	*A*_lift-off_/Å^2^	*A*_π=10mN m_^−1^/Å^2^	*A*_π=30mN m_^−1^/Å^2^	Cs^−1^_max_/mN m^−1^
**DOPC**				
Water	112.9 ± 1.8	85.3 ± 1.5	62.6 ± 0.9	88 ± 4
10^−5^ mol L^−1^ amantadine	146.7 ± 1.7	103.6 ± 0.1	69.5 ± 0.6	58 ± 1

**DMPS**				
Water	77.5 ± 0.5	43.5 ± 0.8	35.4 ± 0.2	293 ± 2
10^−5^ mol L^−1^ amantadine	114.8 ± 4.5	77.6 ± 1.5	33.1 ± 0.1	186 ± 8

**PI**				
Water	111.1 ± 5.7	79.9 ± 2.4	52.2 ± 3.7	54 ± 11
10^−5^ mol L^−1^ amantadine	156.5 ± 7.5	110.5 ± 3.8	57.1 ± 2.7	41 ± 1

**DOPC : DMPS : PI (50 : 35 : 15)**				
Water	90.7 ± 4.0	68.9 ± 3.3	49.7 ± 2.5	105 ± 5
10^−5^ mol L^−1^ amantadine	115.8 ± 2.2	84.5 ± 1.0	59.1 ± 0.7	75 ± 0

The presence of amantadine in the water subphase induced changes in the shape of the isotherms obtained for each system. In the case of the DOPC monolayer, a shift of the isotherms toward larger molecular areas and a decrease in the maximum compressibility modulus can be observed (Fig. 2A). The monolayer becomes more liquid and the distances between lipids increase as a result of interactions between the phospholipids and amantadine. In the presence of the drug, the *C*_s max_^−1^ value decreases to 58 mN m^−1^ (Table 1), which suggests that liquid-expanded (LE) phase organization of the DOPC monolayer is even more pronounced. Changes observed in the DOPC monolayer upon amantadine addition are consistent with previous literature reports: according to Duff *et al.*, amantadine alters the arrangement and translational movement of DOPC molecules within the layer by disrupting lipid–lipid interactions.^21^ Notably, DOPC is composed of two unsaturated alkyl chains, which create space allowing amantadine to locate between lipid molecules. Additionally, some attractive electrostatic interactions between the negatively charged phosphate group of the DOPC polar head and the amantadine amino group, which at pH 7.4 is cationic (p*K*_a_ = 10.6),^24^ are also possible, although due to the net zero charge of the polar head of DOPC these interactions may not be dominant.

Interestingly, interactions between DMPS molecules in the single-component monolayer and amantadine caused a shift of the isotherm toward larger molecular areas, but only at low surface pressures (Fig. 2B). Additionally, a change in the shape of the isotherm concerning its characteristic phase transition at around π = 5 mN m^−1^ is also observed. The fluidizing effect of amantadine on the DMPS monolayer is manifested by a significant decrease in the *C*_s max_^−1^ value to 186 mN m^−1^, which indicates the change of the phase of DMPS monolayer from solid to liquid-condensed (Table 1). Due to the net negative charge of DMPS polar headgroup, cationic amantadine exhibits stronger interactions with the lipid monolayer than with zwitterionic DOPC, due to much stronger attractive electrostatic interactions.^10^ At higher surface pressures, distances between surrounding lipid molecules decrease as amantadine screens the repulsive electrostatic interactions between DMPS headgroups. It may be supposed that amantadine is expulsed from the monolayer and remains within the polar headgroup region. However, drug molecules cause greater fluidization of DMPS monolayer, as evidenced by *C*_s_^−1^-π plots.

Similarly, the PI monolayer exposed to amantadine also showed an increased fluidity due to lipid–drug interactions, which can be observed as a shift of the isotherm toward larger molecular areas and a slightly reduced *C*_s max_^−1^ value reflecting a liquid character of the layer (Fig. 2C and Table 1). Amantadine interacts through attractive electrostatic interactions with negatively charged phosphate group of PI molecule. Additionally, the large size of PI headgroup and its soy extract composition with varying alkyl chain lengths and degrees of saturation, provide space for localizing small amantadine molecules within the layer, facilitating their incorporation into the hydrophobic core of the layer.

The effect of amantadine on the ternary DOPC : DMPS : PI (50 : 35 : 15) monolayer reflects, to some extent, the influence of the drug on the single components (Fig. 2D). The isotherm is shifted towards larger areas per molecule suggesting amantadine incorporation into the layer both due to the electrostatic interactions with negatively charged polar heads of PS and PI components and the ability of the mixed layer to accommodate drug molecules possibly penetrating deeper into the hydrophobic core. However, further compression leads to the partial collapse of the DOPC and PI components concomitant with the expulsion of amantadine from the monolayer, since the isotherms on pure water subphase and subphase with amantadine merge after the partial collapse. To gain more detailed information on the interaction between mixed lipids and amantadine, the excess area for these systems at 1–45 mN m^−1^ surface pressure range was calculated (Fig. S1). For lipid layer formed on a pure water subphase *A*^exc^ is negative across the entire surface pressure range, indicating thermodynamically favourable attractive intermolecular attractions in the mixed layer compared to the interactions between the single components.^66^ Adding amantadine to the subphase resulted in more negative *A*^exc^ values up to 20 mN m^−1^, which is explained by amantadine molecules presence close to the interface and their screening of possible electrostatic repulsions between the lipid heads. At higher surface pressures *A*^exc^ reaches positive values suggesting repulsive interactions between molecules and possible phase separation induced by amantadine.^67–69^

**Fig. 3:**
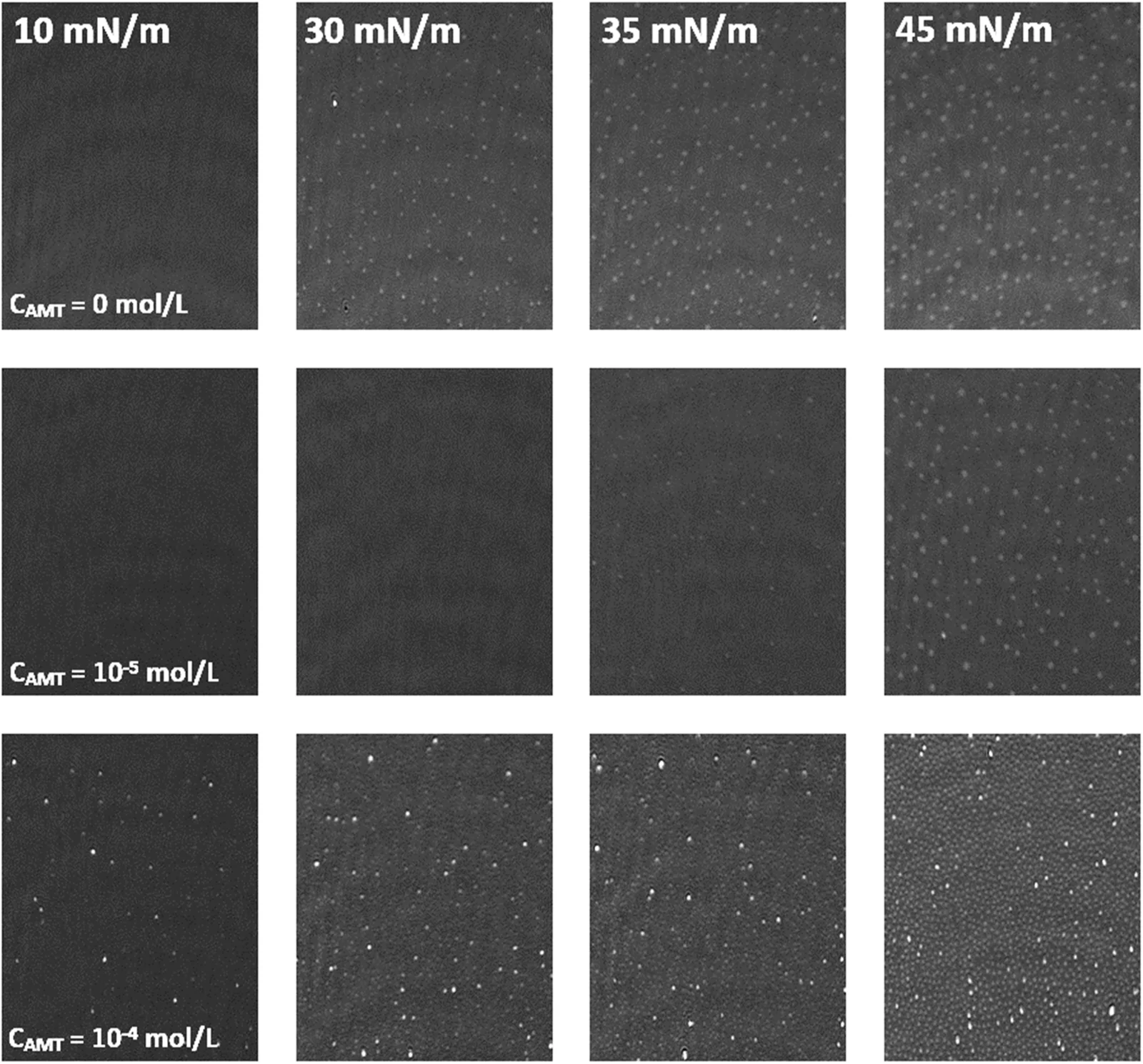
BAM images obtained at selected surface pressures for the monolayers of the mixed model of SARS-CoV-2 composed of DOPC : DMPS : PI (50 : 35 : 15) (upper panel) model membrane exposed to 10^−5^ mol L^−1^ and 10^−4^ mol L^−1^ amantadine (lower panels).

To visualize the changes in the morphology of the ternary monolayer during compression on a pure water subphase and on a water subphase containing amantadine, a series of BAM images was obtained at different surface pressures (Fig. 3). In the case of DOPC : DMPS : PI (50 : 35 : 15) monolayer formed on the pure water subphase, the first small domains can be observed at a surface pressure of 30 mN m^−1^. Further compression leads to domain growth while maintaining their circular shape, although their sizes remained small even at the maximum surface pressure. The addition of amantadine at a concentration of 10^−5^ mol L^−1^ resulted in a delayed domain formation, with domains becoming poorly visible at 35 mN m^−1^. At later stages of compression, their sizes were comparable to those of domains formed in the drug-free environment. According to Wu *et al.*, amantadine at low concentrations induces reorganization of surrounding lipid molecules by disrupting their hydrogen bonds and electrostatic interactions resulting in a final state, where amantadine is present as monomers within the layer.^7^ Due to small changes in the morphology of the ternary monolayer in the presence of 10^−5^ mol L^−1^ amantadine, an additional series of BAM images was also captured for a higher concentration of the drug (10^−4^ mol L^−1^). The observed modifications in the morphology of the model layer were more pronounced, since the first bright domains appeared at a surface pressure of 10 mN m^−1^. With further compression the increase in their number was observed and small darker domains also became visible. At the highest surface pressure, a dense distribution of coexisting bright and darker circular domains was seen. This behaviour may be related to the tendency of amantadine to form aggregates within lipid monolayers at high concentrations,^7,70^ which significantly hinders lipid organization and may lead to phase separation. This would explain the presence of two types of domains (brighter and darker) in the monolayer in the presence of 10^−4^ mol L^−1^ amantadine. The described effects are probably driven by attractive electrostatic interactions between positively charged amantadine and negatively charged DMPS and PI.

In order to further investigate the thermodynamic properties of the ternary DOPC : DMPS : PI monolayer including possible aggregate formation, the cyclic compression and expansion of the ternary monolayer was conducted on a water subphase in the absence and in the presence of amantadine at a concentration of 10^−5^ mol L^−1^ (Fig. 5A). The key thermodynamic parameters: the free energy of hysteresis (Δ*G*^hys^), the entropy of hysteresis (*T*Δ*S*^hys^), and the enthalpy of hysteresis (Δ*H*^hys^) were calculated (Table 2). The addition of the drug caused the appearance of hysteresis in the isotherms, and the change in thermodynamic behaviour is reflected in the calculated parameters. More negative values of Δ*G*^hys^ suggest the retention of a certain amount of free energy during cyclic compression and expansion.^32,33^ In the case of *T*Δ*S*^hys^ a more negative value in the presence of amantadine indicates the formation of more compact domains with a higher organization of molecules, which may result from the presence of enthalpically favourable interactions between molecules. This interpretation is supported by the more negative value of the enthalpy of hysteresis (Δ*H*_hys_) for the cycles recorded in the presence of the drug. These conclusions are in agreement with previous assumptions regarding phase separation induced by the presence of amantadine.

**Table 2:** Thermodynamic functions of hysteresis: the free energy of hysteresis (Δ*G*_hys_), the configurational entropy of hysteresis (*T*Δ*S*^hys^), and the enthalpy of hysteresis (Δ*H*^hys^) calculated between π = 1 mN m^−1^ and π = 30 mN m^−1^ for model SARS-CoV-2 lipid envelope formed on pure water subphase and subphase with 10^−5^ mol L^−1^ amantadine

Subphase	Δ*G*^hys^/kcal mol^−1^	*T*Δ*S*^hys^/kcal mol^−1^	Δ*H*^hys^/kcal mol^−1^
Water	−0.10 ± 0.04	−0.43 ± 0.17	−0.53 ± 0.21
10^−5^ mol L^−1^ amantadine	−0.23 ± 0.01	−0.90 ± 0.06	−1.13 ± 0.06

**Fig. 4:**
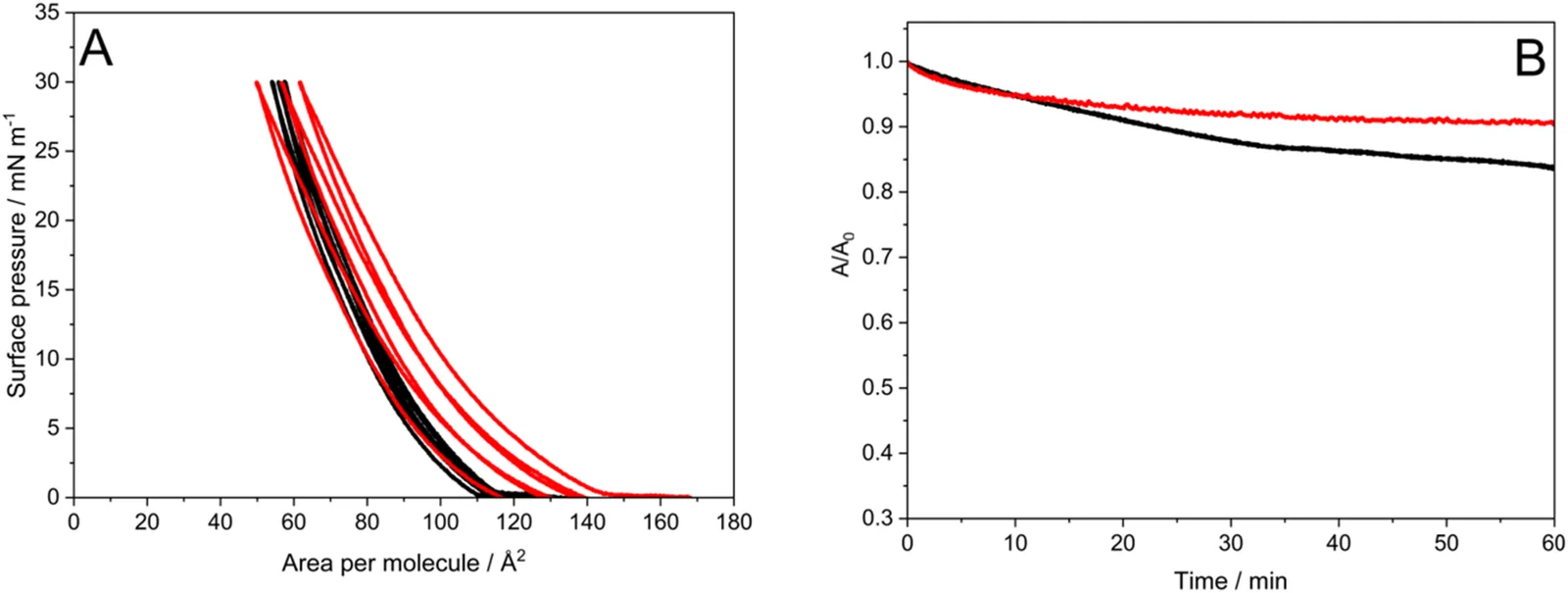
(A) Compression-expansion cycles of the model SARS-CoV-2 lipid envelope formed on pure water subphase (black) and subphase with 10^−5^ mol L^−1^ amantadine (red); (B) changes of area in time of the model SARS-CoV-2 lipid envelope compressed to 30 mN m^−1^ while maintaining the constant surface pressure in time.

In addition, time-dependent surface area measurements were also performed at a constant surface pressure of 30 mN m^−1^, selected to ensure that the properties of the monolayer correspond to those of biological bilayer membranes.^34,35^ The presence of amantadine resulted in an increase in the area per molecule in time compared to the drug-free system within 60 minutes from the start of the experiment when the surface pressure was kept constant at 30 mN m^−1^ (Fig. 4B). Even though the extent of the increase is rather small, this result confirms drug incorporation into the DOPC : DMPS : PI monolayer in time. This small difference may be due to the relatively small size of the drug molecule, which cannot induce a significant increase in the area. However, the observed increase in the area was subsequently examined using 3D systems.

### 3.2. Interactions of amantadine with vesicles as 3D models of viral lipid envelopes

**Fig. 5:**
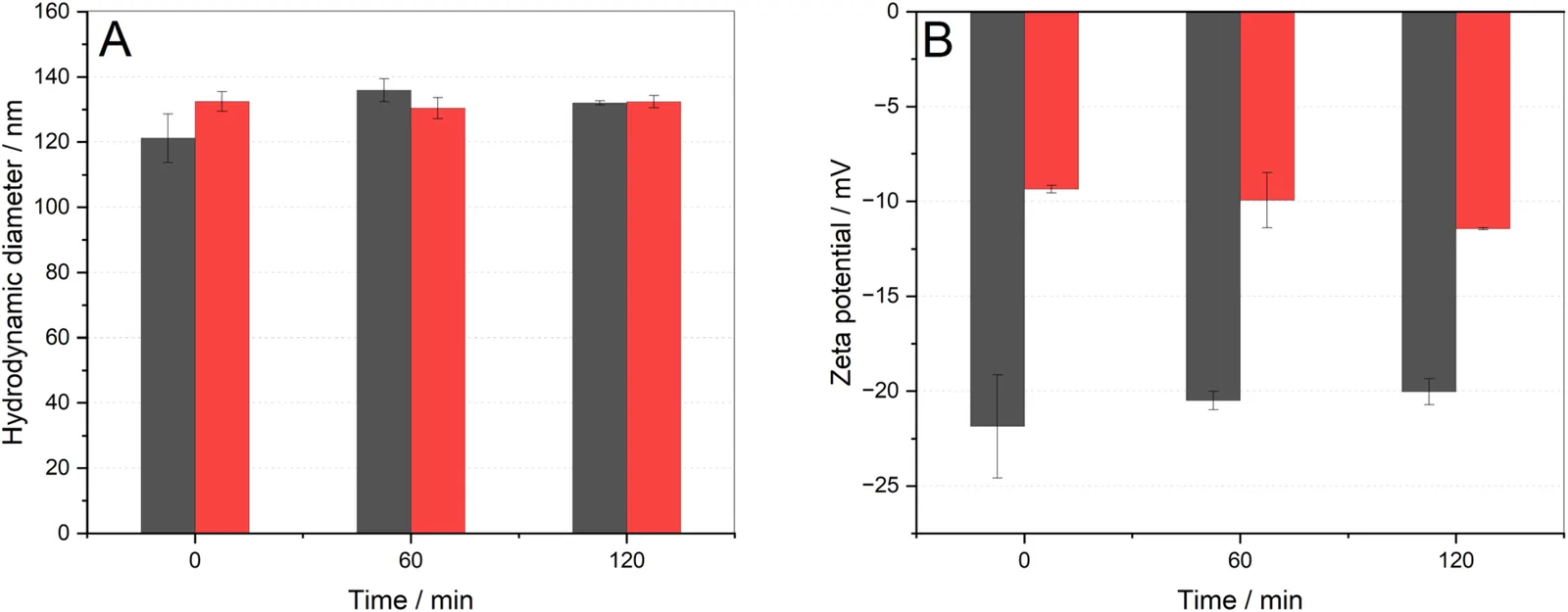
Size distribution of particles in PBS buffer (black) and PBS buffer containing 10^−5^ mol L^−1^ amantadine (red) measured by DLS: (A) changes of hydrodynamic diameter in time; (B) changes of zeta potential in time.

In order to further explore the possible incorporation of amantadine into the lipid layers, 3D models of the viral lipid envelope were employed. Liposomes composed of the same mixture of phospholipids as in the case of monolayer studies: DOPC : DMPS : PI (50 : 35 : 15) were prepared according to the Bangham method (hydration of thin lipid film) to obtain simplified models of SARS-CoV-2 lipid envelope.^26^ The liposomes were investigated in PBS buffer (pH 7.4) in order to ensure their stability. During the first hour of the experiment, the lipids within the liposomes reorganized, as reflected in slight increase in their hydrodynamic diameter (Fig. 5A and Table S1). It can be explained by the presence of two negatively charged lipids in the bilayer, which can interact by repulsive electrostatic interactions hindering the stabilization of their structure. At later stages of the experiment, the diameter values stabilized, reaching 136 nm and 132 nm after 60 and 120 minutes from the start of the measurements, respectively. It should be noted that these values are comparable to the size of the actual SARS-CoV-2 virions.^71,72^ The addition of amantadine at a concentration of 10^−5^ mol L^−1^ led to an increase in the hydrodynamic diameter of the investigated liposomes to 132 nm at the early stages of the experiment, which may suggest changes in the organization of the lipids forming the bilayer due to the interactions with amantadine. However, the hydrodynamic diameter of liposomes exposed to amantadine did not change in time (Table S1). Therefore, the overall effect of amantadine on the hydrodynamic diameter of liposomes is limited.

Interestingly, the effect of the drug on the zeta potential is quite different (Fig. 5B and Table S1). Firstly, it should be noted that the zeta potential values of liposomes in pure PBS buffer remained almost stable throughout the experiment, starting at −22 mV and reaching −20 mV after 120 minutes. This behaviour is expected due to the relatively high density of negative charges on the liposome surface in an environment containing counterions present in the buffer. The addition of amantadine resulted in an increase in the zeta potential to −9 mV at the beginning and −12 mV at the end of the experiment, while achieving even less pronounced fluctuations of the zeta potential in time compared to the drug-free system. Such a significant change indicates the presence of positively charged amantadine molecules close to the liposome surface, leading to partial screening of the negative surface charges.

**Fig. 6:**
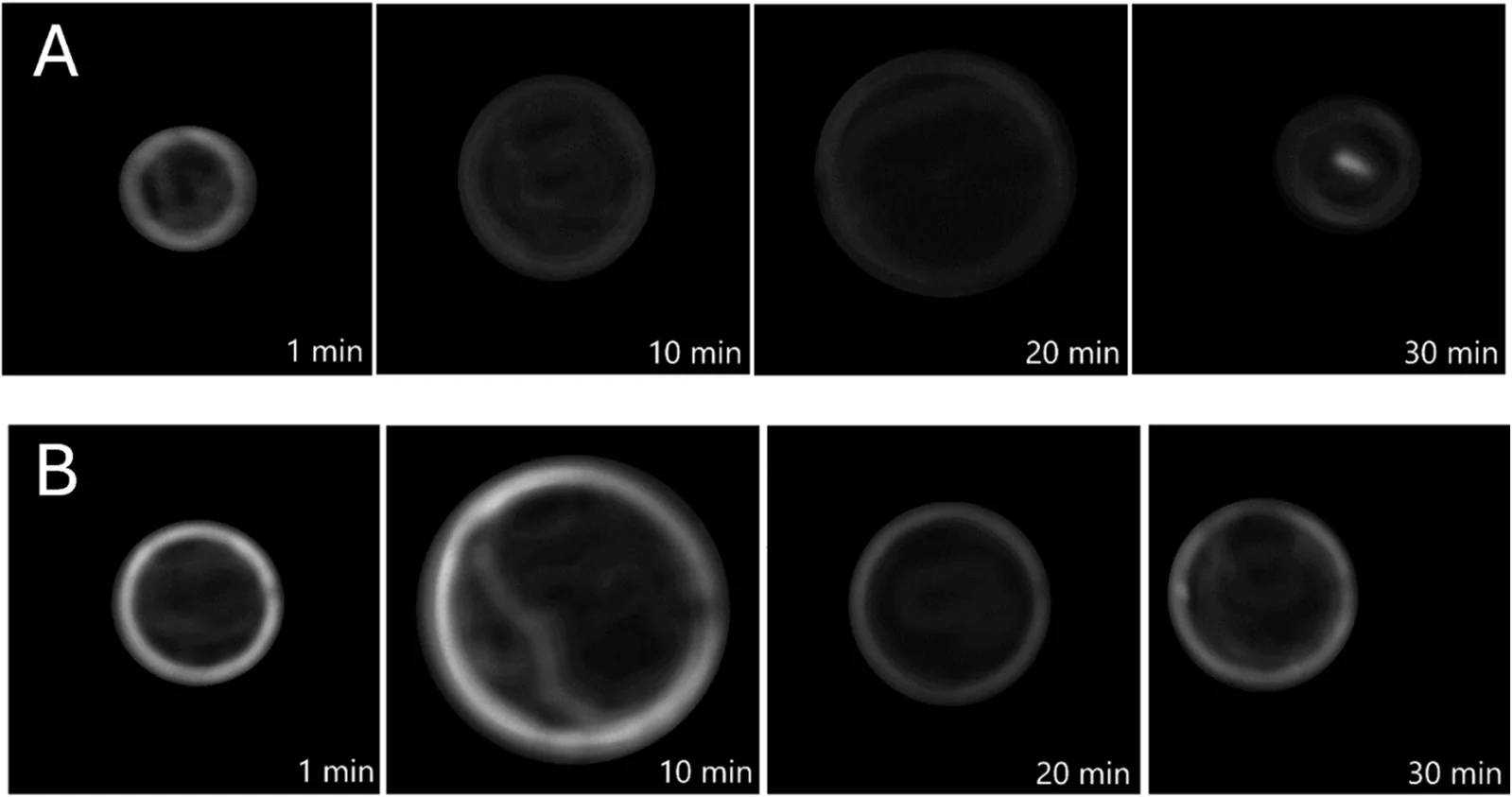
GUVs of the ternary model of SARS-CoV-2 (DOPC : DMPS : PI 50 : 35 : 15) lipid envelope in (A) PBS buffer; (B) PBS buffer with 10^−5^ mol L^−1^ amantadine visualized by fluorescence microscopy.

To better visualize the mechanisms of interaction between amantadine and curved lipid bilayers mimicking the SARS-CoV-2 virus lipid envelope, giant unilamellar vesicles (GUVs) were prepared. Due to their size, GUVs can be observed using fluorescence microscopy if fluorescently labelled lipids are introduced into the bilayer. The vesicles were monitored in a PBS buffer environment in the absence and in the presence of amantadine at a concentration of 10^−5^ mol L^−1^ for 30 minutes (Fig. 6). In the buffer environment, the fluorescence intensity of the selected fluorescent probe decreases over time, reaching its maximum one minute after the start of the experiment. The addition of amantadine resulted in an increase in the fluorescence intensity of the probe during the whole experiment compared to the drug-free system but no shape fluctuations were noted. The observed changes confirm the presence of interactions between amantadine and the lipids forming the bilayer.

### 3.3. Molecular dynamics simulations

#### 3.3.1. Overview of monolayer organization and amantadine distribution

**Fig. 7:**
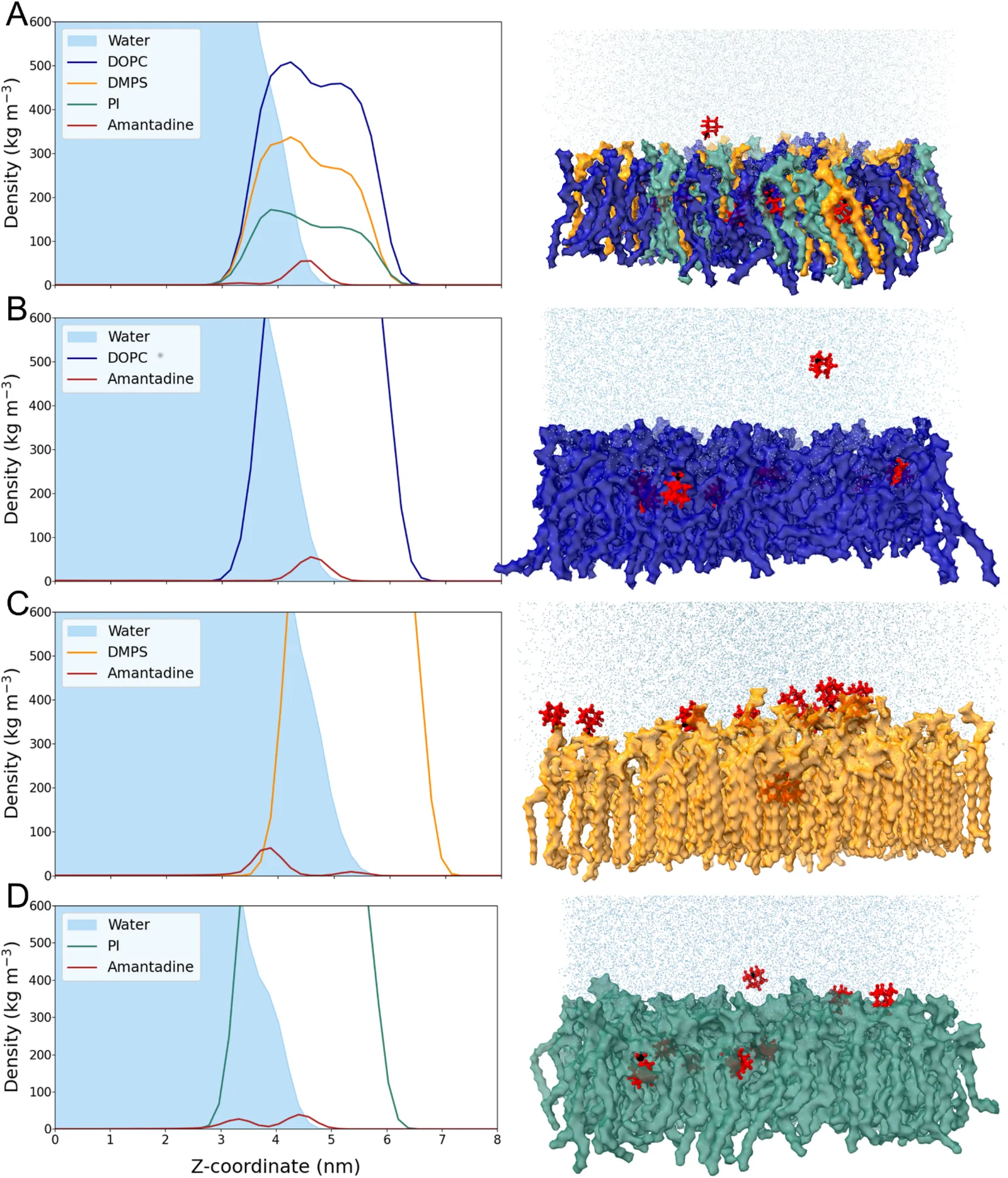
Mass density profiles and representative snapshots, after 400 ns MD simulations, of amantadine at lipid monolayers. Left panels: density profiles of amantadine with (A) ternary DOPC/DMPS/PI (50 : 35 : 15) monolayer, single-component (B) DOPC, (C) DMPS and (D) PI monolayers. Right panels: corresponding molecular-surface renderings showing representative amantadine molecules (red). Lipid surfaces are coloured as follows: DOPC (dark blue), DMPS (orange) and PI (green).

Molecular dynamics (MD) simulations were used to complement the experimental findings by offering molecular-level insight into the localization and penetration of amantadine within lipid monolayers. Pronounced differences are observed among the single-component lipid monolayers (Fig. 7). In the case of DMPS, which adopts a gel-like phase under the applied conditions, amantadine does not penetrate into the monolayer interior. Instead, the drug remains predominantly in the aqueous phase, localizing near the hydrophilic headgroup region. This behaviour is consistent with the tightly packed nature of the gel phase, which limits the availability of free volume within the lipid matrix and thus hinders molecular insertion. In contrast, for the DOPC monolayer, a strong penetration of amantadine into the hydrophobic interior is observed. Density profiles reveal substantial overlap between the drug and the lipid tail region, indicating favourable insertion into the more fluid and loosely packed DOPC environment. The PI monolayer exhibits intermediate behaviour. The density distribution profiles show two distinct maxima for amantadine, corresponding to partial penetration into the monolayer. One population remains associated with the interfacial region near the headgroups, while the second penetrates deeper toward the lipid tails. This bimodal distribution is also evident in the simulation snapshots, confirming the coexistence of surface-associated and partially inserted drug states. This behaviour is consistent with the distinct phase states of the investigated lipids, as DMPS exhibits a gel–fluid transition temperature close to physiological conditions (*T*_m_ ≈ 35–37 °C), whereas DOPC (*T*_m_ ≈ −17 °C) and PI (*T*_m_ ≈ −10 to −5 °C) remain in a fluid phase under the applied conditions, facilitating partial or full penetration of amantadine.

For the mixed lipid monolayer, which no longer exhibits gel-phase characteristics, amantadine is able to penetrate into the monolayer interior. Compared to the DMPS system, the mixed composition displays increased structural disorder and reduced packing constraints, facilitating drug insertion. These observations suggest that the phase transition from a gel-like to a more fluid state leads to layer loosening, thereby lowering the energetic barrier for penetration. Overall, the results demonstrate that lipid composition and phase behaviour play a key role in modulating amantadine localization and penetration at lipid interfaces.

#### 3.3.2. Analysis of the drug–lipid interactions

**Fig. 8:**
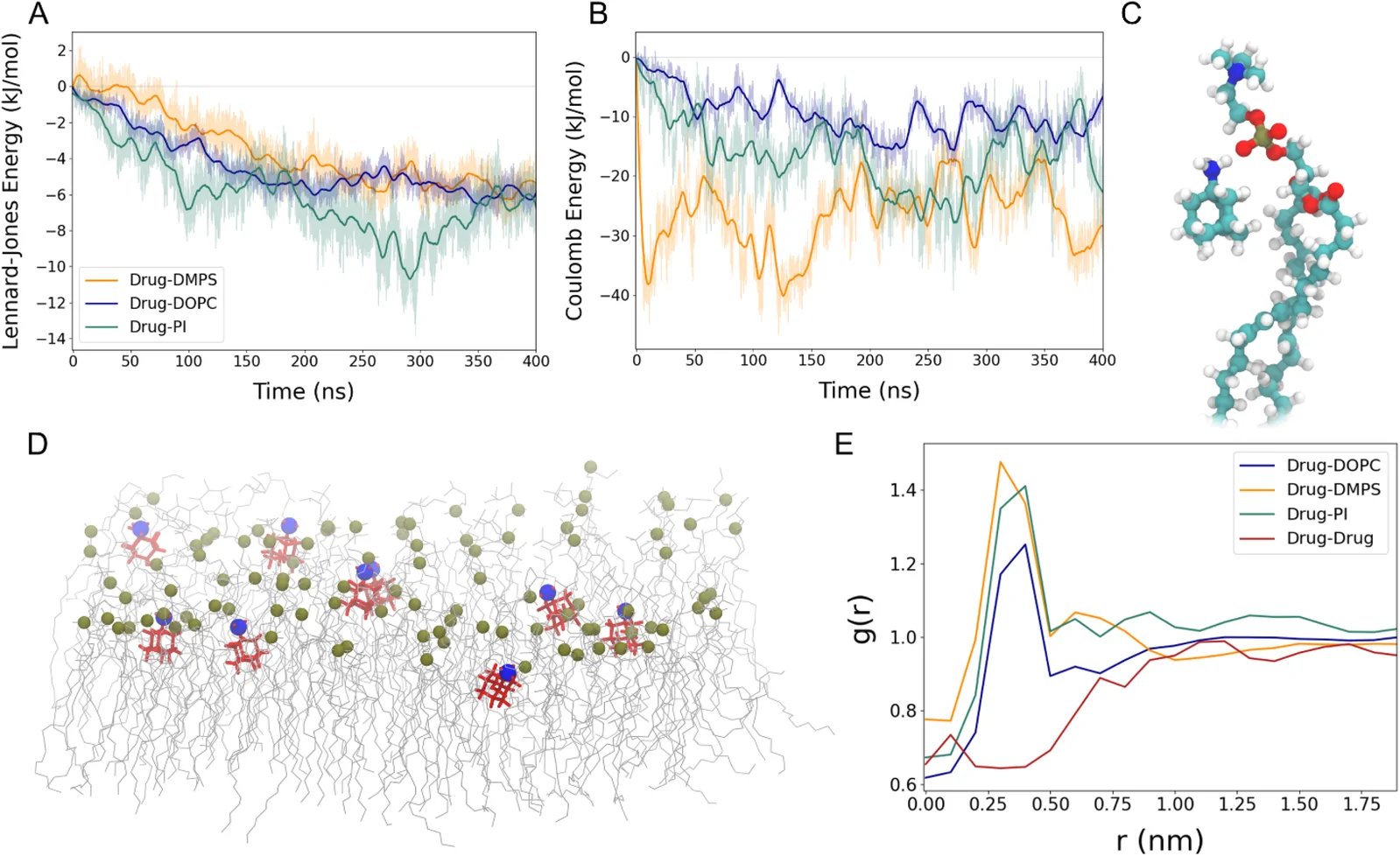
(A and B) Time series of short-range interaction energies between amantadine and each lipid of the ternary monolayer: (A) van der Waals (*via* Lennard-Jones potential) and (B) Coulomb energy. Computed energies were normalized by the number of specific lipids; the legend presented on A applied to B as well. (C) Zoom image of amantadine-DOPC preferred conformation. (D) DOPC : DMPS : PI (50 : 35 : 15) monolayer surface snapshot showing the distribution and orientation of amantadine molecules at the interface; amantadine shown in red with its N atom highlighted in blue, and lipid P atoms highlighted in brown. (E) Lateral radial distribution functions, *g*(*r*), computed for ternary monolayer, between the charged amine group of amantadine and the P atoms in lipid headgroup.

To further rationalize the observed behaviour, next we examined the molecular mechanisms underlying amantadine–lipid interactions and their impact on monolayer organization. Electrostatic interactions were found to dominate amantadine association with the monolayers across all lipid compositions (Fig. 8), indicating the strongest interactions for DMPS (Fig. 8E), followed by PI, with still pronounced interactions observed for DOPC. Despite its zwitterionic nature, amantadine preferentially interacts with the negatively charged phosphate group rather than the choline moiety of DOPC, as shown in Fig. 8C. Van der Waals interactions between the drug and lipids are weaker in comparison to electrostatics, however, not negligible. The strongest interactions were observed for PI lipid, probably due to its large headgroup.

Beyond static interaction strengths, the simulations reveal a dynamic and heterogeneous interaction landscape. Upon penetration into the monolayer, amantadine forms transient interactions with neighbouring lipids, including DOPC and PI, mediated by a combination of electrostatic and van der Waals contributions. The relative involvement of individual lipid species in amantadine coordination fluctuates over time, with intervals dominated by interactions with DMPS alternating with configurations in which interactions with PI or DOPC become more prominent. This dynamic exchange indicates that amantadine does not adopt a single, well-defined strong binding mode, but rather samples multiple local environments within the monolayer, reflecting the intrinsic fluidity and compositional heterogeneity of the system.

Lateral radial distribution functions (Fig. 8E) are in line with the observed trends in the electrostatic interactions, showing that amantadine slightly prefers DMPS and PI over the DOPC. Additionally, *g*(*r*) calculated between amantadine itself shows no aggregation behaviour, which can be also observed in the snapshot in Fig. 8D. It is worth noting, however, that these observations are valid for this particular concentration of amantadine in the monolayer and do not exclude possible aggregation at significantly larger concentrations.

The simulations further indicate a preferential orientation of amantadine at the interface (Fig. 8D), with its charged amine group directed toward the aqueous phase, as clearly visible in the representative simulation snapshots. This orientation is electrostatically favorable, stabilizing the drug near the lipid headgroups and preventing the charged group from penetrating deeply into the hydrophobic region.

## 4. Discussion

The combined approach involving 2D and 3D models of SARS-CoV-2 viral lipid envelope supplemented with MD simulations allowed us to explain in more detail the mechanisms of action of the antiviral agent amantadine with such models and identify the driving forces responsible for the interactions. Langmuir monolayer studies revealed that the drug penetrates both the ternary monolayer and the single-component layers and the effect depends on both the composition of the headgroup of the lipid, namely its net charge, and the organization of the layer determined by the presence of unsaturated alkyl chains in the hydrophobic part. In the case of DOPC (zwitterionic) and PI (negatively charged polar head) layers, which form membranes of a liquid-expanded character, amantadine incorporation into the layer was observed, which resulted in the shift of the isotherm toward the larger areas per molecule. Regarding monolayers formed by DMPS, which is characterized by a net negative charge of the polar part and long, saturated acyl chains resulting in the formation of well-ordered, tightly packed monolayer, amantadine interacted mostly at lower surface pressures corresponding to the less organized monolayer. Further compression led to the expulsion of the drug from the monolayer, since the isotherm in the presence of amantadine intersects that of the pure lipid, resulting in a lower area per lipid molecule in the presence than in the absence of the drug. This may indicate that there is a loss of lipid from the monolayer upon compression, which may be attributed to the formation of drug-lipid aggregates. This phenomenon has previously been observed for DMPS monolayers interacting with anticancer drugs.^73^ Such a different behaviour may be explained by much stronger electrostatic interactions between positively charged amantadine and negatively charged polar heads of DMPS, which prevents amantadine from effectively penetrating the condensed layer. Interestingly, the electrostatic interactions do not block the drug from penetrating more deeply the less-organized phospholipid monolayers such as PI.

These observations are further supported by the results of MD simulations, since the analysis of mass density distributions reveals lipid-dependent differences in amantadine localization: in gel-phase DMPS monolayers the drug remains confined to the interfacial region near the headgroups, whereas in fluid DOPC and PI monolayers, and in the mixed systems, amantadine partially penetrates between lipid headgroups and the upper segments of the hydrophobic chains. This interfacial localization is accompanied by a well-defined orientational preference, with the charged amine group exposed toward the aqueous phase. In the mixed system, amantadine engages in transient interactions with multiple lipid species, dominated by electrostatic interactions with negatively charged phosphate groups and supplemented by weaker van der Waals interactions with lipid tails. Despite the zwitterionic character of DOPC, its phosphate group contributes significantly to drug coordination, providing a molecular explanation for the pronounced changes observed in Langmuir monolayer isotherms. The interaction pattern remains highly dynamic, with amantadine remaining dispersed and continuously exchanging local lipid environments.

Moreover, the excess area calculations for the ternary monolayer indicate a destabilizing effect of amantadine at higher surface pressures, suggesting repulsive interactions and phase separation. This conclusion is further supported by the more negative values of thermodynamic functions of hysteresis pointing to the formation of irreversible aggregates within the mixed layer containing amantadine upon compression–expansion cycles. Additionally, the interactions of amantadine with model ternary lipid layers in time were also observed for monolayers formed at the air–water interface. When compressed to a surface pressure of 30 mN m^−1^, at which the monolayer organization closely resembles that of a biological bilayer while maintaining a constant target surface pressure, the ternary monolayers exhibited the increase in the area per molecule in time compared to the drug-free system. This result indicates drug incorporation into the DOPC : DMPS : PI monolayer over time, which was confirmed by MD simulations.

Such time measurements were also conducted for 3D models of phospholipid bilayers – liposomes. It was shown that despite the attractive electrostatic interactions between positively charged drug and negatively charged phospholipids present in the outer leaflet of the bilayer, the liposome size has not been significantly affected by amantadine. On the other hand, the repulsive interactions between the negatively charged polar heads of lipids leading to an increase in the diameter for liposomes in pure PBS buffer are reduced by the presence of positively charged amantadine, resulting in a more stable size of the liposomes in time. Therefore, the electrostatic interactions between amantadine and lipids may lead to the incorporation of small drug molecules into the lipid bilayer probably within the polar headgroup region.^70^ At the same time, the unsaturated alkyl chains of DOPC and PI, combined with the large size of PI headgroups creating space for the drug, allow amantadine incorporation into the bilayer without any dramatic changes in liposome hydrodynamic diameter.^21,24^

The location of the drug in the polar headgroup region of the bilayer may be additionally supported by the observed increase in the zeta potential of liposomes. The simulations also confirm a preferential orientation of amantadine at the interface (Fig. 8D), with its charged amine group directed toward the aqueous phase, as clearly visible in the simulation snapshots. This orientation is electrostatically favourable, stabilizing the drug near the lipid headgroups and preventing the charged group from penetrating deeply into the hydrophobic region. Importantly, such an orientation provides a molecular-level interpretation of the experimentally observed changes in the *ζ*-potential upon amantadine adsorption on liposomes, as the exposed charged group directly contributes to the interfacial electrostatic environment. It is also consistent with the results of the study by Konstantinidi *et al.*, who showed that amantadine incorporates into DMPC liposome bilayer with its amino group oriented toward the polar headgroup region and its hydrophobic backbone directed toward the membrane's hydrophobic core.^70^

Conclusions drawn for the 3D liposomal models were also supported by the visualisation of the GUVs by fluorescence microscopy. The addition of amantadine resulted in an increase in the fluorescence intensity of the probe throughout the whole experiment compared with the drug-free system. The observed changes are consistent with the presence of interactions between amantadine and the lipids forming the bilayer. However, no changes in vesicle shape were observed, which excludes the presence of significant curvature tension that could arise from the accumulation of large amounts of drug molecules, which have difficulty in incorporating into the outer leaflet. The amantadine–lipid interactions appear to be relatively mild, suggesting that the lipid organization adapts to accommodate the incorporation of drug molecules, probably due to the specific characteristics of the lipids (presence of unsaturated alkyl chains and the large size of PI headgroup). These observations are again supported by the MD simulation results suggesting that amantadine does not adopt a single, well-defined binding mode, but rather samples multiple local environments within the monolayer, reflecting the intrinsic fluidity and compositional heterogeneity of the system.

## 5. Conclusions

In this study, we investigated interactions between the antiviral drug amantadine and the main phospholipid components of the SARS-CoV-2 viral lipid envelope to better understand the potential of amantadine to alter physicochemical properties of the virion envelope. We examined single-component monolayers (DOPC, DMPS, PI) and a ternary monolayer (DOPC : DMPS : PI in a 50 : 35 : 15 molar ratio) as a simplified but accurate model of the SARS-CoV-2 lipid envelope. Langmuir isotherms revealed that amantadine interacts both with single components and the ternary monolayer and the attractive electrostatic interactions play a crucial role in these systems. However, the fluidity of the monolayers is also important, since it allows deeper drug incorporation and further disruption of lipid organization. These observations were confirmed by MD simulation results. BAM images of the lipid envelope model under the influence of amantadine revealed concentration-dependent effects such as delayed lipid domain formation or aggregate formation, which affects later phase separation. We also demonstrated that amantadine incorporates into DOPC : DMPS : PI monolayer in time. Liposome studies using DOPC : DMPS : PI lipid mixture showed amantadine incorporation into the lipid bilayer, stabilization of hydrodynamic diameter and screening of negatively charged lipid headgroups, increasing liposomes' zeta potential. GUV examination further confirmed the easy process of amantadine incorporation including lipid reorganization while maintaining liposome spherical shape.

Despite numerous reports demonstrating amantadine ability to change lipid membrane physicochemical properties,^74,75^ a very limited number of studies has examined its effects on multicomponent models of viral lipid envelopes including the presence of negatively charged lipids and differing lengths and saturation of acyl chains. In our work, we investigated interactions between amantadine and phospholipids to gain deeper insight into the fundamental mechanisms of its effect on SARS-CoV-2 virus lipid envelope, showing some limited selectivity for anionic lipids constituting the model envelope. However, our results highlight the interplay between electrostatic attractions and fluidity and organization of all lipids constituting the model layer. Even though the antiviral activity of amantadine is not only based on its ability to interact with the lipid envelope and therefore the drug by itself might not be the best candidate for this kind of antiviral therapy, our results provide a physicochemical description of the mechanisms governing antiviral agent-viral lipid envelope interactions. Such a fundamental understanding of these mechanisms may be helpful in the development of novel potential antiviral agents for antiviral therapies focusing on changing the stability of viral lipid envelopes, affecting viral protein function by changing the properties of the lipid environment or by inducing changes in the membrane fusion process.

## Conflicts of interest

There are no conflicts of interest to declare.

## Supplementary Material

RA-016-D6RA02249A-s001

## Data Availability

Data are available in the open repository (https://doi.org/10.58132/YCGEH4). Supplementary information (SI) is available. See DOI: https://doi.org/10.1039/d6ra02249a.
