# Supplementary material for: Amantadine-induced reorganization of model SARS-CoV-2 lipid envelopes
Source: RSC Adv. 2026 Jul 21. Online ahead of print. doi: 10.1039/d6ra02249a (PMC13387093; doi:10.1039/d6ra02249a)
Supplement: RA-OLF-D6RA02249A-s001 [file RA-OLF-D6RA02249A-s001.pdf]

Supplementary Information (SI) for RSC Advances.
This journal is © The Royal Society of Chemistry 2026

SUPPORTING INFORMATION

Amantadine-induced reorganization of model SARS-CoV-2 lipid envelopes.

Marta Mierzejewska[1], Izabella Leszczyńska[2], Gabriela Węsierska[1], Piotr Batys[2], Dorota Matyszewska[1*]

[1]*University of Warsaw, Faculty of Chemistry, Biological and Chemical Research Centre, Żwirki i Wigury 101, 02089 Warsaw, Poland*

[2]*Jerzy Haber Institute of Catalysis and Surface Chemistry, Polish Academy of Sciences, Niezapominajek 8, 30239 Krakow, Poland*

Interactions between the components of a lipid mixture can be described based on their miscibility, which is determined from the compression characteristics of single-component monolayers and their mixed films.[1,2] This analysis is based on the theoretical area of the mixed layer ($A^{id}_{1..N}$) and the excess area (A^{Exc}) which are calculated according to the following equations:

$$A^{id}_{1..N} = \sum_N^1 A_i X_i \qquad (1)$$

$$A^{Exc} = A_{1...N} - A^{id}_{1...N} \qquad (2)$$

where A_i is the molecular area of the individual component monolayers at a given surface pressure, and X_i is their molar fraction in the mixed layer. The difference between the area per molecule for mixed monolayer ($A_{1...N}$) at specific surface pressure and the theoretical area per molecule ($A^{id}_{1..N}$) for the same surface pressure is defined as A^{Exc}, as it is shown by Equation 2.

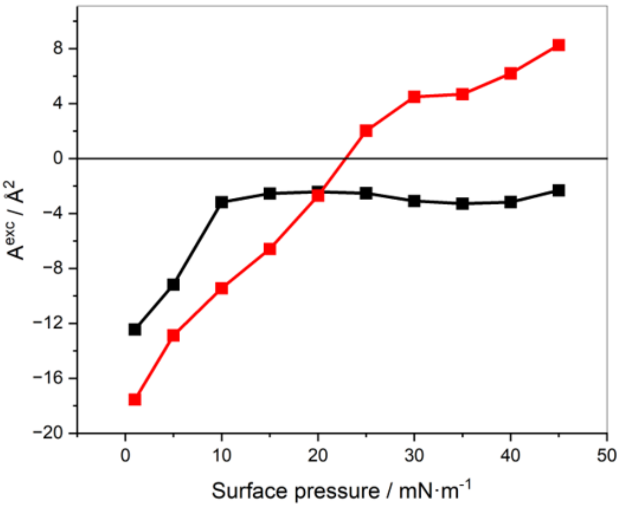

Figure S1 Excess area calculated for the mixed models at different surface pressures for DOPC:DMPS:PI 50:35:15 monolayers on a pure water subphase (black) and subphase containing 10^{-5} mol/L amantadine (red).

Table S1 Characteristic parameters of DOPC:DMPS:PI 50:35:15 liposomes in PBS buffer in the absence and presence of amantadine in the concentration 10^{-5} mol/L.

	0 min		*60 min*		*120 min*	
	d / nm	*ζ / mV*	*d / nm*	*ζ / mV*	*d / nm*	*ζ / mV*
PBS	121 ± 7	-22 ± 3	136 ± 4	-21 ± 0	132 ± 1	-20 ± 1
10^{-5} mol/L amantadine	132 ± 3	-9 ± 0	130 ± 3	-10 ± 0	132 ± 2	-12 ± 0

References:

1 K. Hac-Wydro and P. Dynarowicz-Łatka, *Biophys. Chem.*, 2006, **123**, 154–161.

2 P. Dynarowicz-Łątka and K. Kita, *Adv. Colloid Interface Sci.*, 1999, **79**, 1–17.
